# Genetic mapping and identification of new major loci for tolerance to low phosphorus stress in rice

**DOI:** 10.1007/s12298-020-00858-3

**Published:** 2020-08-17

**Authors:** Abdul Malik Solangi, Hira Khanzada, Ghulam Mustafa Wassan, Adnan Rasheed, Ayaz Ali Keerio, Majeeduddin Solangi, Saba Khanzada, Muhammad Faheem, Jianmin Bian, Xiaohua Pan, Rui Cai Han, Xunfeng He, Ziming Wu

**Affiliations:** 1 Key Laboratory of Crop Physiology, Ecology and Genetic Breeding, Ministry of Education, College of Agronomy, Jiangxi Agricultural University, Nanchang, 330045 Jiangxi People’s Republic of China; 2 Faculty of Crop Production, Sindh Agriculture University Tando Jam, Sindh, Pakistan

**Keywords:** Rice, Backcross recombinant inbreed lines, Single nucleotide polymorphism, QTL analysis, Seedling traits, Low phosphorus

## Abstract

Phosphorus (P) is one of the essential macronutrients for rice. In this study, we used 120 rice backcross recombinant inbred lines (BRILs) derived from a cross *indica cv.* Changhui 891 and *japonica cv.* 02428. To elucidate the genetic control of P deficiency tolerance in rice, we have used high quality SNPs bin markers to identify some important loci underlying phosphorus deficiency. The bin map was generated which includes 3057 bins covering distance of 1266.5 cM with an average of 0.41 cM between markers. Based on this map, 50 loci, including four novel loci, *qSL*-*3*, *qRL*-*11*, *qSDW*-*1*, *qRDW*-*1* with phenotypic variance 23.26%, 12.06%, 9.89% associated with P deficiency-related seedling traits were identified. No significant QTLs was found for root length under P+, shoot fresh weight P− and root length, shoot fresh weight for P+, P− and their ratio respectively. Root fresh weight, and root dry weight were strongly correlated to each other, and QTLs for these variables were located on the same chromosome 1 at the same region. Notably, 3 pleiotropic regions is the pioneer of our study, and these regions would facilitate map-based cloning to expedite the MAS selection for developing low phosphorous tolerant varieties. This study not only improves our knowledge about molecular processes associated with P deficiency, but also provides useful information to understand the genetic architecture of low phosphorous tolerance.

## Introduction

Phosphorus (P) is an essential macro-element for the plant growth and development. It is associated with biological components and soil chemicals that make it important in growing plants (Richardson et al. 2009). It endorses plant growth from root development, early flowering, and seed ripening. Mainly, it is imperative at the early plant growth stages as the plant is recovering from the transplanting shock (Dobermann 2000). However, phosphorus deficiency is a major obstacle for plant development and growth. About half of the agricultural land in Asian, African, and South American countries are facing phosphorus deficiency (Lynch 2011). Moreover, it engenders a series of physiological and molecular processes that led to severe yield losses (Dobermann 2000; Ismail et al. 2007). Besides aforementioned, it is the secondary cause of the low soil pH which forces restrictions in root development, even though there are high concentrations of iron aluminum (Ismail et al. 2007).

Rice (*Oryza sativa* L.) is an essential food as a diet for humans more than half of the world population. Majority of the human population directly or indirectly depends on the rice crop for their daily energy needs (Elert 2014). To meet the availability of rice for the large human community, the rice production needs to be improved by 0.6 to 0.09% in a year (Vinod and Heuer 2012). Like all other cereals, rice also requires a sufficient amount of phosphorus for vigorous and healthy plant growth and seed setting to achieve high yield. Generally, rice response to phosphorus is less studied than response to the nitrogen. Moreover, phosphorus significantly stimulates root enlargement into the new plant, capable to increase the absorbance capacity of nutrients from the soil (Dobermann 2000).

In rice, phosphorous deficient resistant varieties could be produced rapidly through the prominent strategy of Marker Assisted Breeding (Alpuerto et al. 2009). Although, genomic technologies, molecular markers and various statistical methods had significantly altered the genetic analysis of plant breeding. These approaches have provided important tools to locate genomic regions underlying abiotic stresses, including phosphorus tolerance. The physical and genomic maps allow the quantitative trait loci (QTLs) with positive/desired effects on the yield (Chao et al. 2015; Kumar et al. 2017). Rapid improvement in whole-genome sequence technology provided an efficient platform for several million single nucleotide polymorphisms (SNPs) across the genome (Jiang et al. 2017). The SNPs with the same genotype in the internal are joined into bins through a sliding window approach (Huang et al. 2009); and these bins could discriminate recombination events across the whole population and could be used as markers of choice for QTL studies in plant species (Wang et al. 2011). The development of high-density genetic maps generated by bin markers has expedited the genetic studies underlying quantitative traits in various crops (Byrne et al. 2013; Jiang et al. 2017; Poland et al. 2012; Sonah et al. 2013; Wang et al. 2015; Xie et al. 2010; Xu et al. 2010). QTL mapping is an essential strategy to assign the positions of the markers associated with the desired traits. The genome mapping can be used for identifying the QTLs associated with traits related to P deficiency tolerance at the seedling stage (Lin et al. 2010; Wissuwa et al. 2002, 1998; Zhang et al. 2018).

To date, many QTLs have been identified across the rice genome associated with low phosphorus tolerance. However, PSTOL1 is a major candidate gene for low phosphorus, is identified on the chromosome 12 of the rice genome, cloned from P-deficiency tolerant rice variety Kasalath (under name Pup1) (Gamuyao et al. 2012; Wissuwa et al. 2002). To investigate the genetic basis of low phosphorus at seedling stage in rice, numerous studies have been conducted and reported low phosphorus tolerant QTLs identified using different populations (Hu et al. 2001; Li et al. 2009; Lin et al. 2010; Ming et al. 2000; Ni et al. 1998). The screening and development of rice varieties that have properties of phosphorus efficiency provide an alternative and promising solution to resolve this problem, this could be achieved by the use of low phosphorus tolerant genotypes as parents in the rice breeding programs on low phosphorous tolerance (Chin et al. 2011; Wissuwa et al. 1998).

The goal of this study was to identify QTLs for low phosphorous tolerance. High resolution QTL mapping has been reported through sequencing based genotyping of 120 rice backcross recombinant inbreed lines (BRILs). We analyzed the main effects and digenic interactions of QTLs for seedling growth traits under low phosphorus and high phosphorus stresses. The QTLs identified in this study will be valuable sources for phosphorus deficiency studies in rice.

## Materials and methods

### Plant population

In this study, BRILs (BC1F6) population consisting of 120 individuals derived from Changhui 891, a leading *indica* restorer line in south China and 02428, a prominent *japonica* wide compatibility variety, were used for QTL mapping for low Phosphorus. The population was developed in the experimental field at Jiangxi Agricultural University in Nanchang, Jiangxi Province, and Linwang, Hainan Province.

### Hydroponic experiment

The experiment was conducted at Key Laboratory of Crop Physiology, Ecology, and Genetics Breeding Of Jiangxi Agricultural University, China. Briefly, 30–40 uniform seeds were selected from 120 BRILs along with their parental lines and surface-sterilized for 20 min in 1% (w/v) sodium hypochlorite solution and then washed with distilled water, then sterilized seeds were used for germination at 28 °C for 48/64 h. After germination, ten seedlings were transplanted into a PCR tube, and transferred in plastic boxes. The box contains either P-deficiency (P-) or P-sufficiency (P+) aerated nutrient solution, formulation of the nutrient solution was, according to (Yoshida et al. 1971), with some modifications. The culture solution was prepared in distilled water. The nutrient solution was renewed weekly, and the normal culture solution was a mixture of (mg/L); Ca (NO_3_)_2_, 24.8 KH_2_PO_4_, 48.2 (NH_4_) _2_SO_4_, 15.9 K_2_SO_4_, 65.9 MgSO_4_ and 59.918.5 KNO_3_ and 1 mL/L MnCl_2_·4H_2_O, CuSO_4_·5H_2_O, H_2_MoO_4_·H_2_O, H_3_BO_3_, ZnSO_4_·7H_2_O and Fe-EDTA. For the low phosphorus treatment, the KH_2_PO_4_ application reduced to 0.32 (mg/L). The pH value of solutions was adjusted to 5.0 by using NaOH or HCl. A randomized complete block design with three replications was used for each experiment. All the lines and their parents were planted randomly. Seedlings were harvested 30 days after planting.

### Phenotypic traits measurements

A total of six seedling traits were measured with three replicates from all of the lines under both levels of phosphorus. The phenotypic data were recorded for all traits for measuring the shoot length (SL) and root length (RL) by using a ruler. To determine the fresh weight, the seedlings were washed with water, and the shoot fresh weight (SFW), root fresh weight (RFW) were noted by using digital weight balance. For dry weight, the roots and shoots were put in craft paper envelope for drying at 65^◦^C for 72 h in an oven. The shoot dry weight (SDW) and root dry weight (RDW) were determined. All the traits were investigated for QTL analysis associated with low phosphorus tolerance, respectively.

### Phenotypic data analysis

Phenotypic data were analyzed using analysis of variance (ANOVA) by Minitab v.18 software (Pennsylvania State University, PA, USA) based on general linear model at *p* < 0.05 level and the means were compared using the Tukey’s honest significant difference (HSD) test. Pearson’s correlation, descriptive statistics, frequency distribution, and box plot analysis were carried out using the SPSS version 20 (SPSS, Chicago, IL, USA). The broad-sense heritability was calculated using the formula: *h*^2^ = Vg (Vg + Ve/r), where, (Vg) genotypic variance, (Ve) error variance and r is replication.

### QTL analysis in BRILs population for six seedling traits

To investigate the QTLs for tolerance to low phosphorus in BRIL population, QTL IciMapping v4.1 software was used for QTL analysis, threshold LOD Value of 2.5 was applied (Bian et al. 2015; Jiang et al. 2017). QTL nomenclature was followed by the method of (McCouch 2008).

## Results

### Phenotypic performance of 120 BRILs under P+ and P− and their ratio

**Fig. 1:**
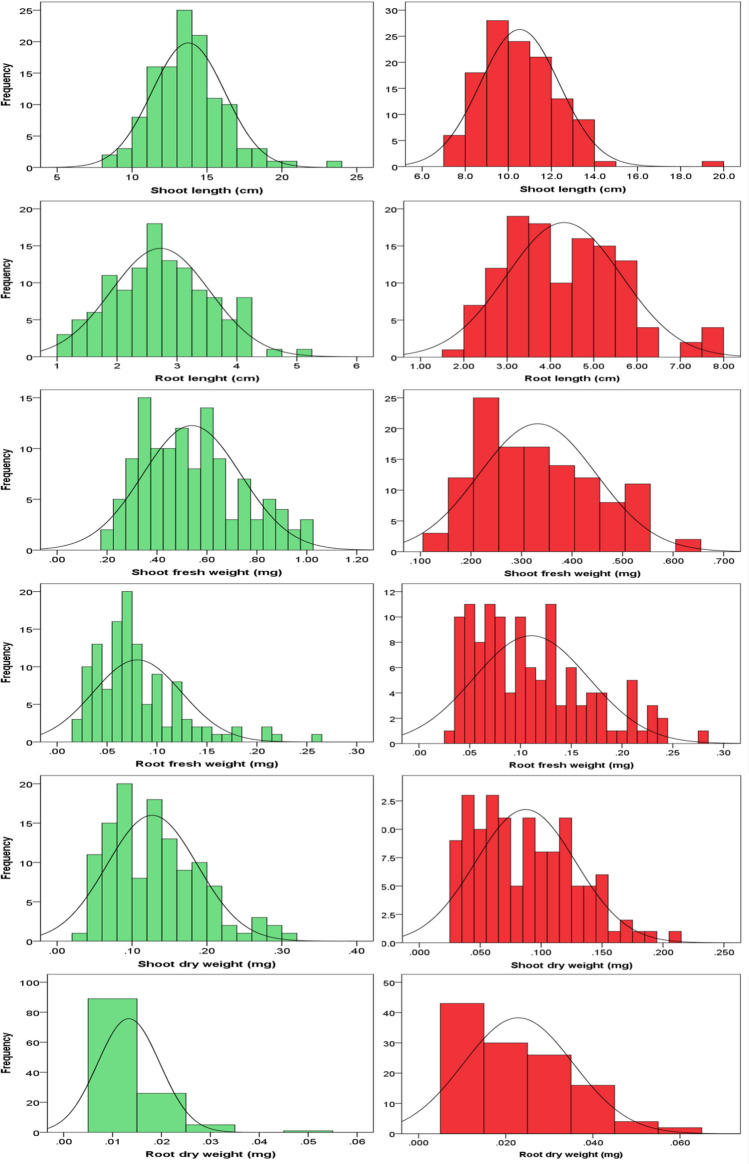
Phenotypic distribution of six seedling traits associated with P + and P − in BRILs. Green color indicate the P + and red color indicate P − , respectively

**Fig. 2:**
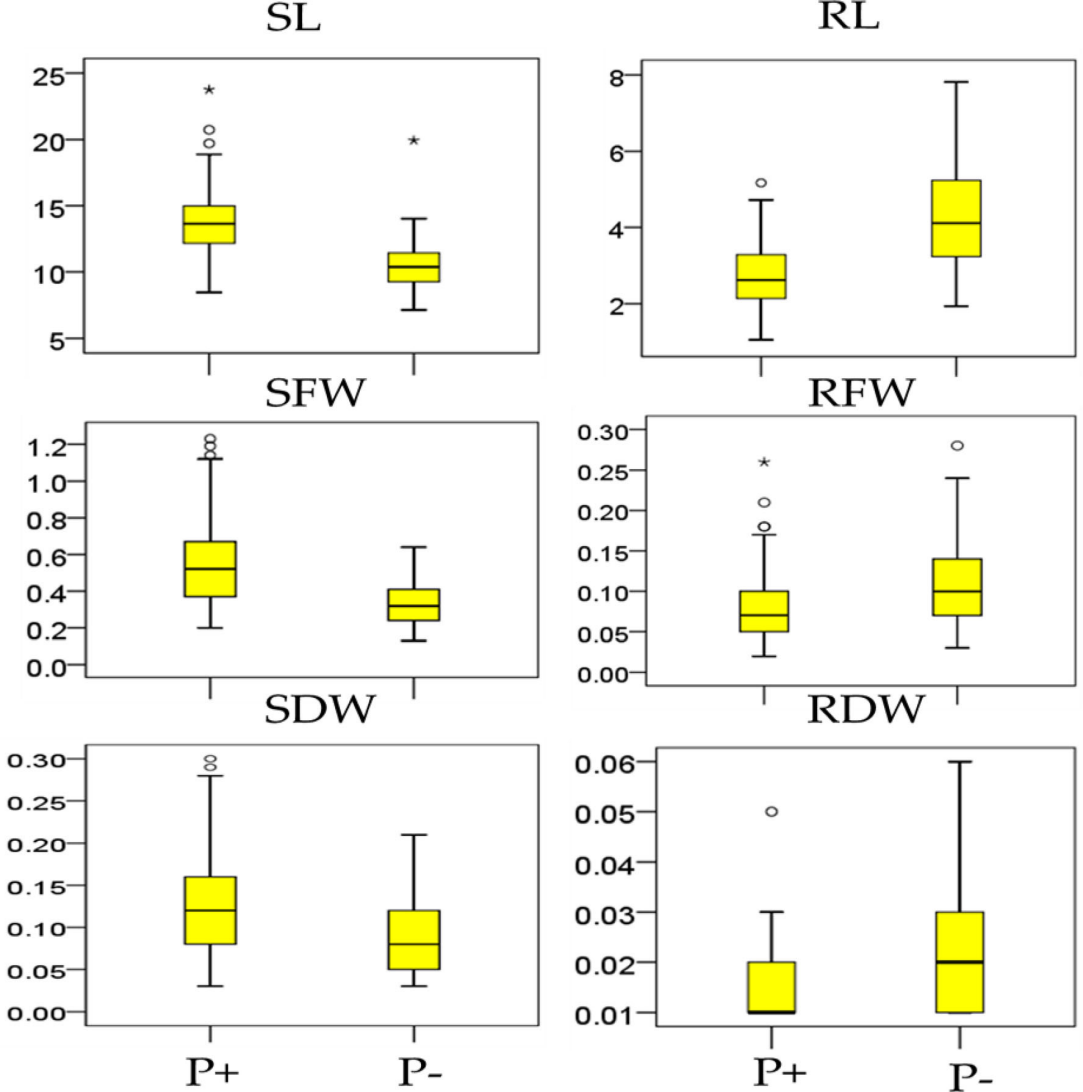
Box plot analysis of six seedling traits under two phosphorus level P + and P − , SL, shoot length, RL, root length, SFW shoot fresh weight, RFW, root fresh weight, SDW, shoot dry weight, RDW, root dry weight, while the circles represent outliers

Analysis of variance and broad-sense heritability among six seedling traits of 120 BRILs under both levels of phosphorus (P+ and P−) are displayed in Table 1. For all the studied traits, analysis of variance showed highly significant difference among the evaluated lines which indicated the presence of large genetic variability. Interaction of lines, treatment, and line x treatment were found highly significant for all studied traits. Mean squares revealed significant variations among BRILs for all seedling traits. Estimates of broad sense heritability were found higher for all six traits, SL (96.71%), RL (82.64%), SFW (96.08%), RFW (87.97%), SDW (87.32%) and RDW (63.81%) indicated that all traits are highly heritable, and selection of these traits would be fruitful for successful breeding. The descriptive statistics for six seedling traits of BRILs under both levels of P+ and P− are shown in Table 2. However, mean values for shoot length (SL), under P+, P− (ranging from 8.47, 23.75 and 7.15, 19.9). Mean performance for root length (RL) under P+, P− varied from 1.05, 5.10 and 1.94, 7.81. For shoot fresh weight, root fresh weight, shoot dry weight and root dry weight mean values under the both experimental conditions (P+, P−) ranged from 0.20, 1.23 and 0.64, 0.11; 0.26, 0.04 and 0.03, 3.05; 0.03, 0.30 and 0.21, 0.04 and 0.01, 0.05 and 0.01, 0.06 respectively. However, the frequency distribution and box plot (Fig. 1 and 2) analysis showed that traits are normally distributed in the population. Skewness and Kurtosis for all six variables in BRILs were less than unit, showed a normal distribution of traits and data could be subjected to Quantitative traits loci analysis.

**Table 1:** Analysis of variance and broad sense heritability of six seedling traits under P + and P − conditions

Traits	SOV	Df	SS	MS	*F*-Value	*P* Value	*h*^2^
SL	Trt	1	1838.62	1838.62	2465.84	0.000	96.71%
	Lines	119	2713.85	22.81	30.59	0.000	
	Trt × Lines	119	724.91	6.09	8.17	0.000	
	Error	478	356.41	0.75			
RL	Trt	1	426.29	426.294	702.68	0.000	82.64%
	Lines	119	416.27	3.498	5.77	0.000	
	Trt × Lines	119	546.35	4.591	7.57	0.000	
	Error	478	289.99	0.607			
SFW	Trt	1	9.2027	9.20272	1586.21	0.000	96.08%
	Lines	119	17.6059	0.14795	25.50	0.000	
	Trt × Lines	119	3.9049	0.03281	5.66	0.000	
	Error	478	2.7732	0.00580			
RFW	Trt	1	0.08147	0.081473	64.39	0.000	87.97%
	Lines	119	1.24264	0.010442	8.25	0.000	
	Trt × Lines	119	0.53082	0.004461	3.53	0.000	
	Error	478	0.60478	0.001265			
SDW	Trt	1	0.08891	0.088911	58.21	0.000	87.32%
	Lines	119	1.43367	0.012048	7.89	0.000	
	Trt × Lines	119	0.29882	0.002511	1.64	0.000	
	Error	478	0.73010	0.001527			
RDW	Trt	1	0.023987	0.023987	211.13	0.000	63.81%
	Lines	119	0.037454	0.000315	2.77	0.000	
	Trt × Lines	119	0.019216	0.000161	1.42	0.006	
	Error	478	0.054306	0.000114			

SL, shoot length, RL, root length, SFW shoot fresh weight, RFW, root fresh weight, SDW, shoot dry weight, RDW, root dry weight, SOV, source of variance, df, degree of freedom, SS, sum of squares, MS, mean squares, Trt, treatment, *h*^2^ heritability

**Table 2:** Descriptive statistics of six seedling traits observed under different P+ , P− conditions

Trait	Trt	Mean	Min.	Max.	SD	CV (%)	Skewness	Kurtosis
SL	P+	13.756	8.4700	23.750	2.43	17.72	0.7678	1.7536
	P−	10.530	7.1500	19.940	1.83	17.43	1.2254	4.2045
RL	P+	2.7238	1.0500	5.1700	0.82	30.23	0.7678	1.7536
	P−	4.2889	1.9400	7.8100	1.34	31.32	0.5070	-0.2600
SFW	P+	0.5584	0.2000	1.2300	0.06	39.60	0.8240	0.3300
	P−	0.3321	0.1300	0.6400	0.11	34.97	0.4759	-0.6464
RFW	P+	0.0803	0.0200	0.2600	0.04	55.06	1.4549	2.5996
	P−	0.1355	0.0300	3.0500	0.27	201.58	10.171	106.08
SDW	P+	0.1269	0.0300	0.3000	0.06	47.58	0.6682	−0.0310
	P−	0.0873	0.0300	0.2100	0.04	47.10	0.5351	−0.3927
RDW	P+	0.0133	0.0100	0.0500	0.006376	47.91	2.4946	8.5151
	P−	0.0229	0.0100	0.0600	0.01	55.09	0.7443	−0.1509

SL, shoot length, RL, root length, SFW shoot fresh weight, RFW, root fresh weight, SDW, shoot dry weight, RDW, root dry weight, Trt. = Treatment, Min. = Minimum, Max. = Maximum, SD = Standard deviation, CV = Coefficient of variance

### Correlation coefficient analysis of six seedling traits

In our study, we examined the phenotypic correlation coefficient analysis among the six seedling traits separately; under both P+, P− level (results are presented in Table 3). Under P+, P− condition, significant correlations were observed for all studied traits, SL, RL, SFW, RFW, SDW, and RDW with few exceptions. The results ranged from 0.14 to 0.84 under P+, and from 0.02 to 0.92 under P- deficient level (Table 3). Highest positive significant correlation under P + 0.8446 was observed between SFW and SDW while under P− highly significant positive correlation 0.92 was recorded among the SDW and SFW. Due to the highly significant correlation among the seedling trait, it might be possible to improve one trait through an indirect selection of other traits.

**Table 3:** Pearson’s phenotypic correlation coefficient among six seedling traits measured under P + and P − condition

Traits	SL	RL	SFW	RFW	SDW	RDW
SL	1	0.3637**	0.4430**	0.2526*	0.3893**	0.2043*
RL	0.2537*	1	0.2545*	0.2838*	0.3748**	0.1479*
SFW	0.6734 **	0.2940*	1	0.7184**	0.8446**	0.4895**
RFW	0.0881^NS^	0.0200^NS^	0.2150*	1	0.6368**	0.5280**
SDW	0.6400**	0.2187*	0.9222**	0.2043**	1	0.5318**
RDW	0.4266**	0.4259**	0.7014**	0.0728^NS^	0.6856**	1

**Significant at *p* < 0. 01 and *Significant at *p* < 0.05. The data above the diagonal correspond to correlations among traits under high P, while the below ones under low P stress. SL, shoot length, RL, root length, SFW shoot fresh weight, RFW, root fresh weight, SDW, shoot dry weight, RDW, root dry weight

### QTL analysis for six seedling root traits under P+ and P− levels and their relative ratio

In our study, we have identified a total of 50 QTLs with significant LOD value for all traits under the two P levels; 21 in P+, 12 in P− and 17 in P+/P− ratio. Among six seedlings traits, no significant QTLs was found for root length under P+, shoot fresh weight P− and root length, shoot fresh weight for P+, P− and their ratio, respectively.

### QTL detection for P+

**Fig. 3:**
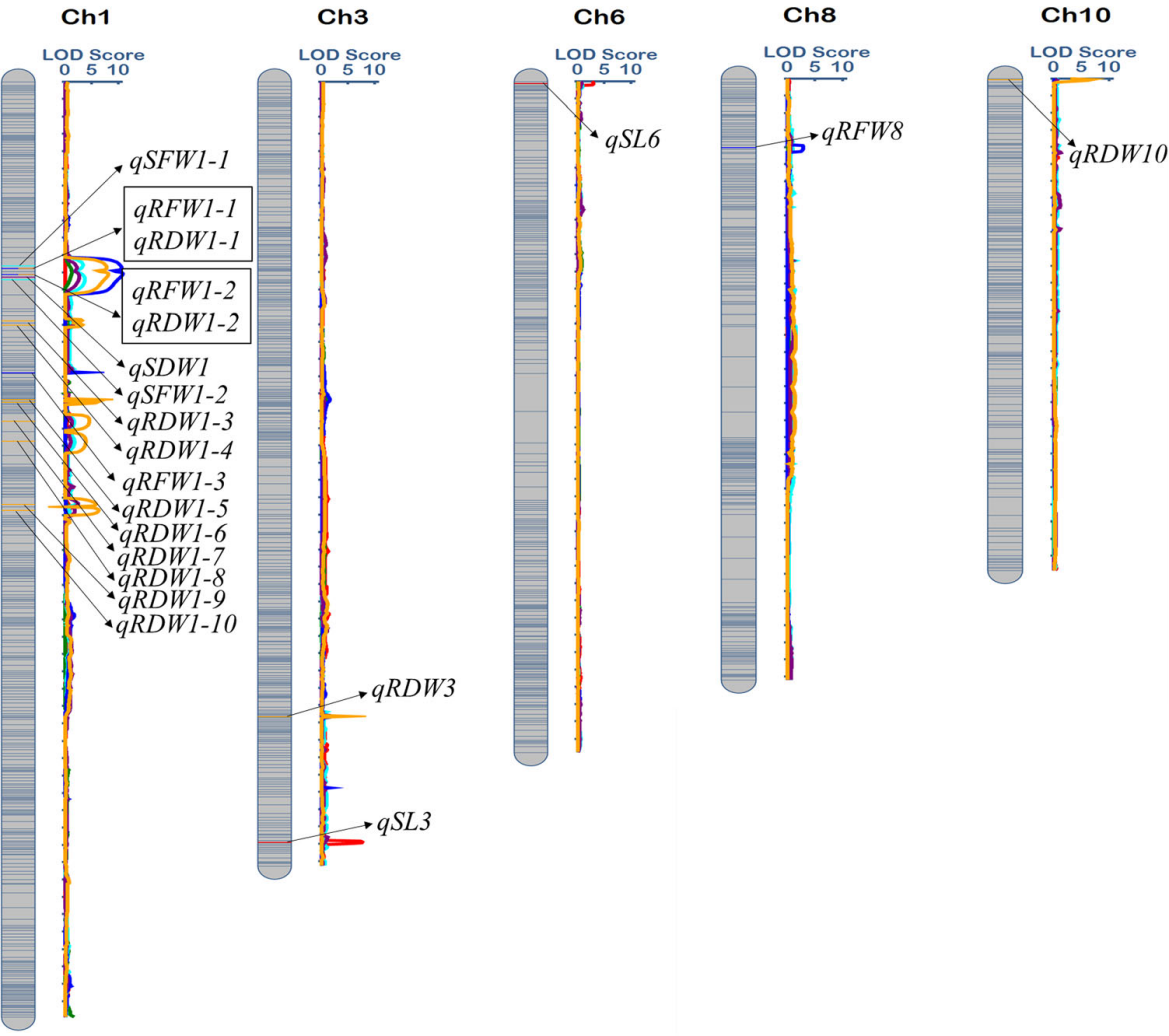
Chromosomal location of putative QTLs for seedling traits associated with P + . The QTLs displayed in square box, indicates the pleotropic QTLs on chromosome 1

A total of 21 QTLs were detected, with LOD value ranging from 2.64 to 10.81 and a portion of variance contributed by these genomic regions varied from 0.74 to 23.26% on the different chromosome for different traits (Table 4 and Fig. 3). Two QTLs of SL were identified on chromosome 3 and 6. QTLs *qSL*-*3* and *qSL*-*6* were mapped between marker 3-305, 3-306 which shows the phenotypic and LOD values of two QTLs were 23.26 and 7.60 and 8.24 and 2.97, respectively. Two QTLs, *qSFW*-*1* was identified for SFW on the same chromosome with different position, between marker 1–72, 1–73 and 1–73, 1–74 with 2.95, 3.68, phenotypic % and 3.32, 3.69 LOD Value, respectively. For RFW, four QTLs, *qRFW*-*1*were mapped. Three on chromosome 1 and one *qRFW*-*8* on chromosome 8, with different positions, phenotypic variance and LOD value of 10.56%, 10.81% and 3.62 and 2.86, respectively. Single QTL, *qSDW*-*1* was mapped on chromosome 1 for SDW between the marker interval 1–73, 1–74 which explained the 6.77% phenotypic variance. A total of 10 QTLs were mapped on chromosome 1 at different positions, with LOD values ranging from 8.09 to 6.25 and these QTLs showed varying degree of their contribution to total phenotypic variance by 0.85% to 0.91%. One QTL, *qRDW*-*3* was mapped on chromosome 3 between the flanking markers of 3–257 and 3-258 for root dry weight securing LOD value 4.66 and portion of phenotypic variance contributed by this QTL was 0.77%. Chromosome 10 harboring the QTL *qRDW*-*10* for root dry weight between the markers 10–1 and 10–2 with LOD value 6.90 and phenotypic variance 1.16%. All traits under P+ showed negative additive effect, which demonstrated that the value of traits had been decreased by these QTLs. Chromosome 1 harboring the QTLs for two different traits on the same position, which is an indication of the strong correlation of these traits, and these traits could be taken to improve high phosphorous tolerance. Two pleiotropic QTLs *qRFW*-*1* and *qRDW*-*1* were identified on the chromosome 1 controlling the RFW and RDW at the same position. Hence, chromosome 1 harboring two pleiotropic QTLs for two traits, which should be a new way to study multiple characters governed by the same locus.

**Table 4:** QTLs identified under P + for six seedlings traits in BRILs

Trait	QTLs	Chr	Position	Left Marker	Right Marker	LOD	PVE (%)	Add	Left CI	Right CI
SL	*qSL*-*3*	3	880.00	bin3-305	bin3-306	7.60	23.26	− 2.72	878.50	881.50
	*qSL*-*6*	6	3.00	bin6-1	bin6-2	2.97	8.24	1.14	1.00	5.50
SFW	*qSFW*-*1*	1	215.50	bin1-72	bin1-73	3.32	2.95	− 0.24	209.00	221.00
	*qSFW*-*1*	1	231.50	bin1-73	bin1-74	3.69	3.68	− 0.23	224.00	242.00
RFW	*qRFW*-*1*	1	218.50	bin1-72	bin1-73	10.56	7.76	− 0.06	211.00	221.00
	*qRFW*-*1*	1	225.50	bin1-73	bin1-74	10.81	7.81	− 0.06	223.00	232.00
	*qRFW*-*1*	1	338.50	bin1-93	bin1-94	3.62	4.14	− 0.07	338.00	339.00
	*qRFW*-*8*	8	83.50	bin8-32	bin8-33	2.86	1.68	− 0.01	81.00	89.00
SDW	*qSDW*-*1*	1	228.50	bin1-73	bin1-74	2.64	6.77	− 0.06	222.00	240.00
RDW	*qRDW*-*1*	1	218.50	bin1-72	bin1-73	7.77	0.90	− 0.00	211.00	221.00
	*qRDW*-*1*	1	225.50	bin1-73	bin1-74	8.09	0.91	− 0.00	223.00	233.00
	*qRDW*-*1*	1	278.50	bin1-77	bin1-78	3.12	0.81	− 0.00	277.00	280.00
	*qRDW*-*1*	1	283.50	bin1-78	bin1-79	3.20	0.81	− 0.00	282.00	286.00
	*qRDW*-*1*	1	369.50	bi1-112	bin1-113	5.38	0.74	− 0.01	369.00	370.00
	*qRDW*-*1*	1	372.50	bin1-114	bin1-115	6.12	0.77	− 0.01	372.00	374.00
	*qRDW*-*1*	1	394.50	bin1-121	bin1-122	4.52	0.88	− 0.00	388.00	403.00
	*qRDW*-*1*	1	417.50	bin1-122	bin1-123	3.85	0.91	− 0.00	410.00	429.00
	*qRDW*-*1*	1	490.50	bin1-142	bin1-143	5.76	0.84	− 0.00	487.00	493.00
	*qRDW*-*1*	1	497.50	bin1-143	bin1-144	6.25	0.85	− 0.00	495.00	502.00
	*qRDW*-*3*	3	735.00	bin3-257	bin3-258	4.66	0.77	− 0.01	734.50	735.50
	*qRDW*-*10*	10	2.00	bin10-1	bin10-2	6.90	1.16	− 0.00	1.00	3.50

SL, shoot length, SFW shoot fresh weight, RFW, root fresh weight, SDW, shoot dry weight, RDW, root dry weight, Chr, chromosome, LOD, logarithm of odds, PVE (%), percent of variance explained (%), Add, Additive effect, CI, confidence interval

### QTL detection for P−

**Fig. 4:**
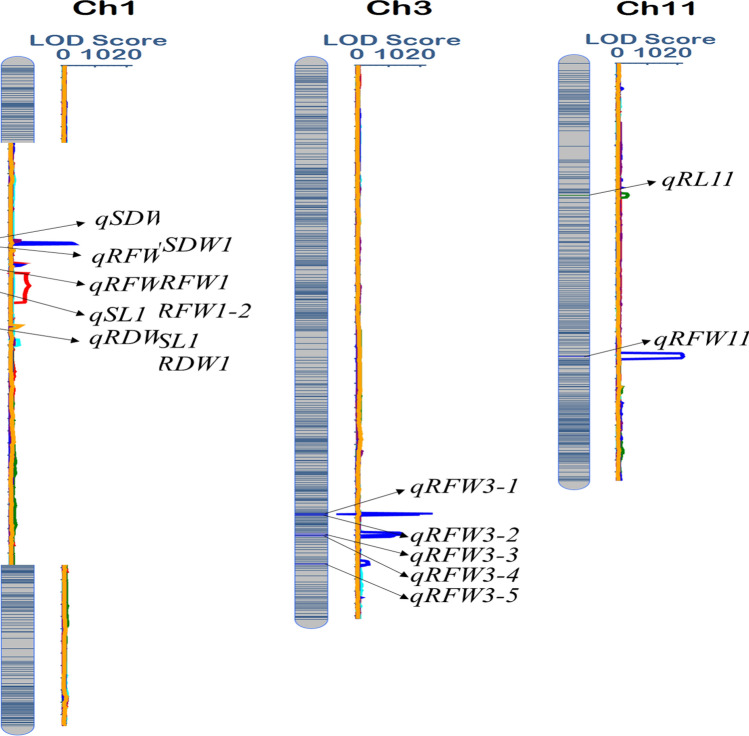
Chromosomal location of putative QTLs for seedling traits associated with low P − stress

Under the low P- condition, a total of 12 QTLs were identified with LOD value of 2.78 to 21.74 with 2.08% to 20.45% phenotypic variation (Table 5 and Fig. 4). Among these twelve QTLs, one QTL, *qSL*-*1* was mapped between the marker 1–122 and 1–123 with highest phenotypic contribution of 20.45% and having 5.78 LOD value. For root length, *qRL*-*11* alone QTL was mapped between the marker 11–55 and 11–56 with the LOD value of 3.30 and 12.06% phenotypic variation. For root fresh weight, eight QTLs were detected; 2 of them were on the chromosome 1 with LOD values ranging from 20.13, 3.40 and 12.06%, 5.82% phenotypic variation explained. Five QTLs were mapped on chromosome 3 and one on chromosome 11. For SDW and RDW, single QTL was detected between marker 1–92–193 with 2.78 LOD score and explained the 9.89% phenotypic variation, while *qRDW*-*1* mapped on the same chromosome among the bins- 1–37, 1–38 which contributed about 10.29% phenotypic variation and has 3.23 LOD value.

**Table 5:** QTLs identified under P- for six seedling traits in BRILs

Trait	QTLs	Chr	Position	Left Marker	Right Marker	LOD	PVE (%)	Add	Left CI	Right CI
SL	*qSL*-*1*	1	406.50	bin1-122	bin1-123	5.78	20.45	− 1.86	396.00	414.00
RL	*qRL*-*1*	11	235.50	bin11-55	bin11-56	3.30	12.06	− 0.87	232.00	240.00
RFW	*qRFW*-*1*	1	338.50	bin1-93	bin1-94	20.13	5.82	− 0.93	336.00	339.00
	*qRFW*-*1*	1	372.50	bin1-114	bin1-115	3.40	2.27	0.58	372.00	374.00
	*qRFW*-*3*	3	735.00	bin3-257	bin3-258	16.8	5.82	− 0.93	734.50	735.50
	*qRFW*-*3*	3	737.00	bin3-258	bin3-259	12.84	5.55	− 0.64	736.50	737.50
	*qRFW*-*3*	3	768.00	bin3-275	bin3-276	13.79	5.91	0.54	765.50	769.50
	*qRFW*-*3*	3	771.00	bin3-276	bin3-277	11.31	5.91	0.54	770.50	772.50
	*qRFW*-*3*	3	817.00	bin3-289	bin3-290	3.40	2.08	0.39	815.50	818.50
	*qRFW*-*11*	11	511.50	bin11-139	bin11-140	21.74	10.74	− 0.74	510.00	515.00
SDW	*qSDW*-*1*	1	333.50	bin1-92	bin1-93	2.789	9.89	− 0.03	332.00	335.00
RDW	*qRDW*-*1*	1	471.50	bin1-137	bin1-138	3.23	10.29	− 0.01	471.00	475.00

SL, shoot length, RL, root length, RFW, root fresh weight, SDW, shoot dry weight, RDW, root dry weight, Chr, chromosome, LOD, logarithm of odds, PVE (%), percent of variance explained (%), Add, Additive effect, CI, confidence interval

### QTLs detected for P+/P− ratio

**Fig. 5:**
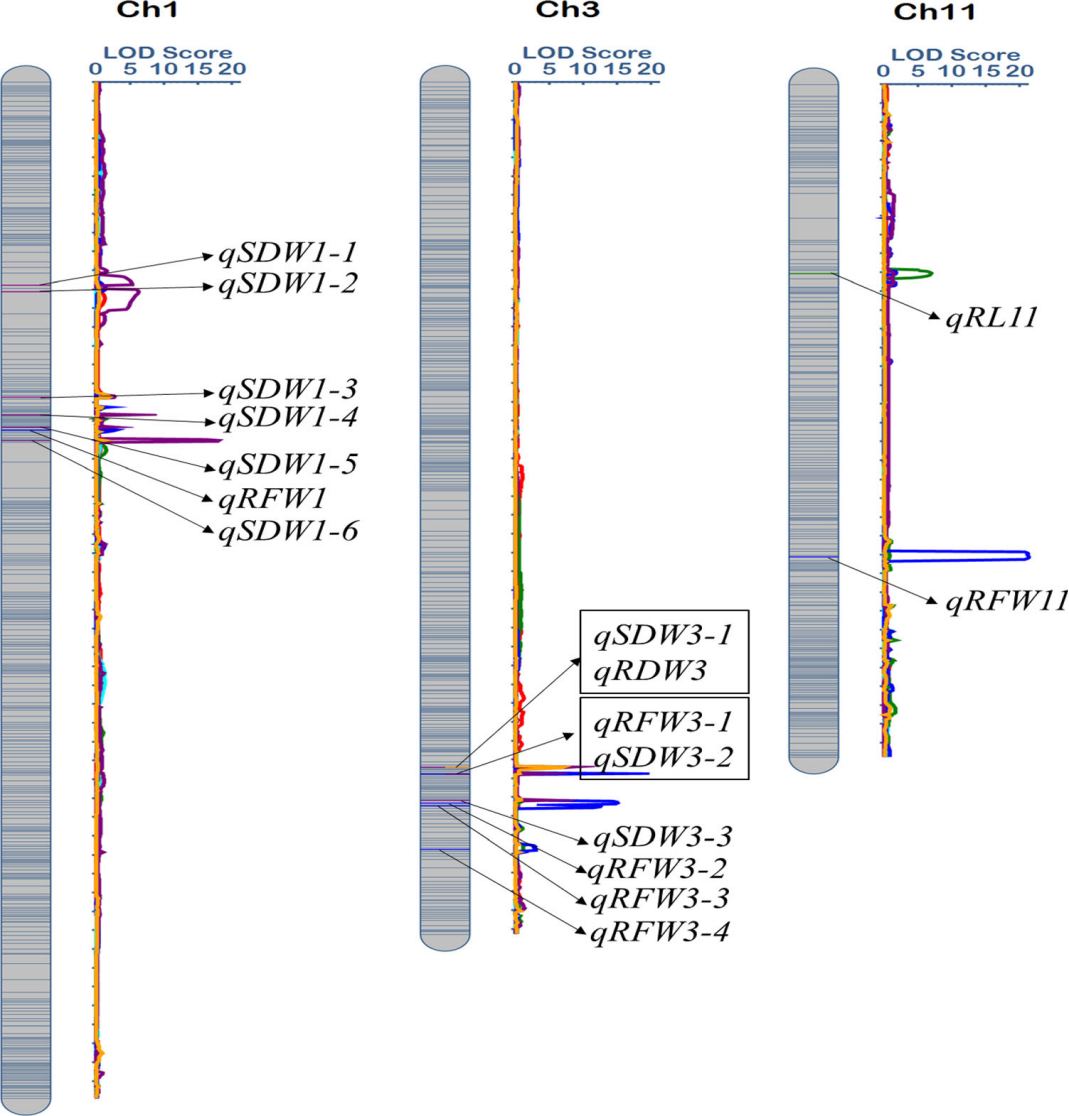
Location of putative QTLs for seedling traits ratio under both level of Phosphorus P +/P − , while QTLs displayed in square box, indicates the pleotropic QTLs

The ratio of P+ and P− is the best strategy to investigate the putative regions involved in governing traits under low and high phosphorous stresses. Only four variables have been noted, holding a varying number of QTLs (Table 6 and Fig. 5). Total of 3 chromosomes was mapped at various regions for a total of 17 QTLs. Root length is a key factor or trait used to measure low phosphorous tolerance. Currently, only one genomic region was mapped at chromosome 11 for SL with 6.87 LOD value and 19.39% of phenotypic fraction contributed by this region. Root fresh weight recorded 6 QTLs on chromosome 1, 3, and 11 with their LOD scores of 2.98–21.25 and range of phenotypic variance showed by these regions was 2.99% to 14.84%. A total of 9 QTLs *qSDW*-*1* and *qSDW*-*3* were distributed on chromosomes 1 and 3 for SDW securing LOD score 5.28–5.49 with 3.32–3.25 portion of phenotypic variation shared by these loci. The genomic region on chromosome 3 harboring the QTL *qRDW*-*3* for root dry weight among the flanking markers 3–254, 3–255. Chromosome 3 harboring one pleiotropic QTL qSDW-3 involved in controlling two traits at the same position and flanked by the same markers.

**Table 6:** QTLs identified under both condition P +/P − for seedling traits in BRILs

Trait	QTLs	Chr	Position	Left Marker	Right Marker	LOD	PVE (%)	Add	Left CI	Right CI
RL	qRL-11	11	210.50	bin11-47	bin11-48	6.87	19.39	− 1.29	208.00	214.00
RFW	qRFW-1	1	372.50	bin1-114	bin1-115	2.98	2.99	18.87	372.00	374.00
	qRFW-3	3	737.00	bin3-258	bin3-259	16.54	8.55	− 22.67	736.50	737.50
	qRFW-3	3	768.00	bin3-275	bin3-276	14.98	8.79	18.85	765.50	769.50
	qRFW-3	3	771.00	bin3-276	bin3-277	12.50	8.79	18.85	770.50	772.50
	qRFW-3	3	817.00	bin3-289	bin3-290	3.01	2.74	12.85	815.50	818.50
	qRFW-11	11	511.50	bin11-139	bin11-140	21.25	14.84	− 24.52	510.00	515.00
SDW	qSDW-1	1	218.50	bin1-72	bin1-73	5.28	3.32	− 0.58	211.00	219.00
	qSDW-1	1	225.50	bin1-73	bin1-74	6.20	3.39	− 0.55	223.00	235.00
	qSDW-1	1	337.50	bin1-93	bin1-94	2.66	1.00	0.48	336.00	339.00
	qSDW-1	1	356.50	bin1-102	bin1-103	5.76	3.25	− 0.62	356.00	357.00
	qSDW-1	1	369.50	bin1-112	bin1-113	2.56	1.00	0.48	369.00	370.00
	qSDW-1	1	383.50	bin1-119	bin1-120	18.00	6.36	− 0.86	383.00	385.00
	qSDW-3	3	730.00	bin3-254	bin3-255	8.62	3.25	− 0.62	729.50	730.50
	qSDW-3	3	737.00	bin3-258	bin3-259	4.58	3.25	− 0.62	736.50	737.50
	qSDW-3	3	765.00	bin3-274	bin3-275	5.49	3.25	− 0.62	764.50	765.50
RDW	qRDW-3	3	730.00	bin3-254	bin3-255	7.16	13.09	− 1.63	729.50	731.50

RL, root length, RFW, root fresh weight, SDW, shoot dry weight, RDW, root dry weight, Chr, chromosome, LOD, logarithm of odds, PVE (%), percent of variance explained (%), Add, Additive effect, CI, confidence interval

## Discussion

Rice growth is severely affected by low phosphorus in rice growing areas. High-resolution mapping population is a basic need for any breeding scheme to identify the regions controlling traits of rice grown under low phosphorus conditions. Many populations such as chromosome segment substitution lines (CSSLs), recombinant inbred lines (RILs), near-isogenic lines (NILs) and introgression lines (ILs) have been widely studied to investigate the genetic analysis for abiotic stresses in crops. However, because of the several limitations, these cause hindrance to examine the results more accurately. CSSLs and NILs were used mainly for studying rice tolerance to low phosphorous, but these populations consume more time and increase labor cost, and these populations are overlooked under such conditions. Currently, we have used a set of 120 BRILs to get a clear clue about the targeted regions on chromosomes. Use of BRIL population gives a comprehensive understanding of QTLs governing traits under different phosphorus levels. Many studies have been conducted earlier to identify the QTLs using different P levels (Anis et al. 2018; Wang et al. 2014).

In the current study, we have evaluated a set of 120 BRILs derived from a cross between *Japonica* (cv.02428) and *Indica* (Changhui/891) background parents using a linkage map constructed by SNP markers. Therefore, we assume this population will serve as a better choice for mapping and studying important quantitative traits in rice. QTL analysis was conducted for six seedling traits under two P levels (P− as deficiency and P+ as sufficiency). An ideal way to measure the tolerance degree is to grow the crops in the best nutrient solution. We have focused on shoot length because of its simple measurement in response to P- stress. Shoot and root traits has been studied earlier for measuring low P- tolerance (Anis et al. 2018; Li et al. 2009). We have identified 3 QTLs *qSL*-*1*, *qSL*-*3*, *qSL*-*6* for shoot length, one in P- and two under P + on the chromosomes number 1 and 3. QTL *qSL*-*1* was marked among the markers 1-22 and 1-23, explained the phenotypic variance of this QTL which was 20.45% while QTLs under normal P level was mapped on chromosome 3 between different bins 3–305, 3–306 and 3–1, 3–2 with 23.26 and 8.24% of phenotypic variation was showed by these QTLs. The QTL *qSL*-*3* showed the highest percentage of variance 23.26%, which showed its larger effect on the shoot length. One of our identified QTL *qSL*-*6* was reported on the same chromosome (Anis et al. 2018; Li et al. 2009; Ming et al. 2000). Another researcher, Yugandhar et al. (2017) identified QTL *qSL*-*1* on chromosome 1 in rice grown under low P similar to our studies.

However, we mapped one QTL on different chromosome 3, differed from their studies, which introduced a new theory of using these regions for detection of more QTLs in the future. However, not any significant QTL for shoot length was detected under P+/P− conditions beside root length, shoot length also considered as an important trait for low phosphorous tolerance. Two QTLs, *qRL*-*11* were detected on the same chromosome 11 at different positions in P- and P+/P− conditions. QTL *qRL*-*11* was marked between 11 and 55, 11–56 bins with 3.30 of LOD value and 12.06% phenotypic variance reported root length along with 6.87% of phenotypic variance, 6.87 LOD value for QTL *qRL*-*11* mapped on same chromosome 11 among the marker 11–47, 11–48 bins under P +/P− levels. Zhang et al. (2006) reported three QTLs for root length, one of them correspond to our identified QTL *qRL*-*11*, but here we mapped one more QTL which might be a novel point to find out the role of root length in low phosphorous response. Under P+, only two QTLs were identified for shoot fresh weight on chromosome 1 with LOD value and phenotypic variance 3.32–3.69% and 2.95–3.68%. No QTLs were detected under P− and P+/P− for SFW.

Our study had showed contrasting results with previously identified QTLs. Moreover, the root fresh weight is also studied in the current experiment to investigate its role in enhancing low P- tolerance in BRILs. Eight QTLs *qRFW*-*1*, *qRFW*-*3,* and *qRFW*-*11* under P-, two on chromosome 1, five on chromosome 3 and one on 11 with LOD values 20.3 to 21.74 and 5.82% to 10.74% of phenotypic variation reported for these. Li et al. (2009) reported two QTLs for root fresh weight with positive additive effect under P-. QTL *qRFW*-*3* identified on same chromosome in our study but here 4 more QTLs were identified for root fresh weight in our BRILs seedling traits on chromosome 3 which would facilitate in increasing attention of researchers to target these regions for unfolding low phosphorous tolerance mechanism. Four QTLs for RFW were identified in P+ with LOD value of 10.56–2.86 and 7.76–1.68% of phenotypic variance. Six QTLs were identified for RFW, one (*qRFW*-1) of them is on the chromosome 1, four (*qRFW*-*3)* on chromosome 3, and one (qRFW-6) on chromosome 6 were detected for RFW with LOD value and phenotypic variation of 2.98–21.25 and 2.99% to 14.84%, respectively. Shoot dry weight plays an important role to measure the degree of tolerance under low phosphorous. Two QTLs *qSDW*-*1* under P− and P+ were mapped on chromosome 1 among the bins 1–92, 1–93 and 2.78 and 9.89, LOD value and phenotypic variation recorded for QTL under P− and 2.64 LOD value and 6.77% of phenotypic variance for QTL detected under P+.

Many researchers in the past conducted QTL analysis and identified QTLs for shoot dry weight. Li et al. (2009) and Ming et al. (2001) evaluated rice DH lines and IL for QTLs detection under low P and identified one QTL on chromosome 12 and one on chromosome 6 different from our results which might be due to use of different breeding lines. Chromosome one harbor more important regions governing shoot dry weight. Map-based cloning of these points could be more fruitful for MAS selection.

Under P− and P+/P−, two QTLs were detected for RDW on chromosomes 1 and 3. LOD values 3.32 and 7.16 and phenotypic variance 10.24% and 13.09% contributed by these QTLs. A total of 12 QTLs based on their position, ten on chromosome 1, one on 3 and one on 10 chromosomes were detected under P+ level. These QTLs LOD values ranged from 7.77 to 6.90, and 0.90%–1.16% of variances were noted. Many studied conducted earlier are an agreement with our findings, and some showed contrast because of the difference in breeding material and solution used. Yugandhar et al. (2017) detected 3 QTLs for root dry weight on chromosomes 3, 5 and 10 with their phenotypic contribution of 10.82–12.52%. QTL detected on chromosome 3 is consistent to our identified QTL *qSDW*-*3*; we have identified one new QTL on chromosome 1 which indicated the possibility of this region to involve in increasing shoot dry weight under P deficiency tolerance.

Quantitative traits loci mapping for tolerance to low phosphorous will be more useful in the identification of important regions to be cloned for tolerance. In our results, more important indicator of low phosphorous tolerance is shoot length. Among these 50 QTLs, *qSL*-*3*, *qRL*-*11* were novel QTLs, which would be important to transfer these regions into BRILs for increasing crop growth efficiency under low phosphorous deficient conditions. Two QTLs *qSDW*-*1* for secondary tolerance indices like shoot dry weight mapped on chromosome 1 at different regions were newly reported QTLs. One QTL for RDW on chromosome one, *qRDW*-*1* is novel QTL, and these traits could also be used for evaluating the degree of low phosphorous tolerance in rice. Many traits showed inter-relationship because of the presence of their QTLs on the same chromosome. Root fresh weight and root dry weight were strongly correlated to each other, and QTLs for these variables were located on the chromosome 1 at same region. Extremely tolerant lines could be used to develop low phosphorous resistant varieties and markers behind these QTLs would be useful to enhance MAS selection for low P tolerance. Effective nutrient solution and nature of population should be more focused to carry out QTL analysis to get insight into low P tolerance mechanism. Identification of 3 pleiotropic QTL, two for SFW and RDW in P+ and one for RFW and SDW in P+ and P− ratio would be a novel point to isolate these regions and investigate the mechanism of these regions to enhance BRILs tolerance.

## Conclusions

Rice is one of the most important crop of the world, and the presence of abiotic stresses are limiting its growth and ultimately effecting its production. Development of varieties resistant to low phosphorous is an urgent need to maintain rice growth on effected soils. A total of 50 QTLs, 21 under P+, 12 under P− and 17 under P+/P− for six seedling traits were detected by evaluating 120 BRILs developed from a cross between *Japonica* (cv.02428) and *Indica* (Changhui) under two levels of phosphorous P+ and P−. Out of 50 QTLs, major QTLs *qSL*-*3*, *qRL*-*11*, *qSDW*-*1*, *qRDW*-*1* involved in response to low phosphorus tolerance were novel QTLs. Root fresh weight and root dry weight were strongly correlated to each other, and QTLs for these variables were located on the same chromosome 1 at the same region. Identification of 3 pleiotropic regions is the novelty of our results, and these would be helpful to enhance tolerance via transformation of this locus into BRIL population. These regions would facilitate map-based cloning to speed up MAS selection for developing low phosphorous tolerant varieties.

## Abbreviations

BRILs: Backcross recombinant inbreed lines
QTLs: Quantitative trait loci
SNP: Single nucleotide polymorphism
cm: Centimeter
cM: Centimorgan
mg: Milligram
SL: Shoot length
RL: Root length
SFW: Shoot fresh weight
RFW: Root fresh weight
SDW: Shoot dry weight
RDW: Root dry weight
P: Phosphorus
MAS: Marker assisted selection
